# Effects of a Voluntary Front-of-Pack Nutrition Labelling System on Packaged Food Reformulation: The Health Star Rating System in New Zealand

**DOI:** 10.3390/nu9080918

**Published:** 2017-08-22

**Authors:** Cliona Ni Mhurchu, Helen Eyles, Yeun-Hyang Choi

**Affiliations:** National Institute for Health Innovation, University of Auckland, Private Bag 92019, Auckland Mail Centre, Auckland 1142, New Zealand; h.eyles@auckland.ac.nz (H.E.); yeunhyang.choi@auckland.ac.nz (Y.-H.C.)

**Keywords:** nutrition, labelling, diet, composition, reformulation, health star rating

## Abstract

Interpretive, front-of-pack (FOP) nutrition labels may encourage reformulation of packaged foods. We aimed to evaluate the effects of the Health Star Rating (HSR), a new voluntary interpretive FOP labelling system, on food reformulation in New Zealand. Annual surveys of packaged food and beverage labelling and composition were undertaken in supermarkets before and after adoption of HSR i.e., 2014 to 2016. Outcomes assessed were HSR uptake by food group star ratings of products displaying a HSR label; nutritional composition of products displaying HSR compared with non-HSR products; and the composition of products displaying HSR labels in 2016 compared with their composition prior to introduction of HSR. In 2016, two years after adoption of the voluntary system, 5.3% of packaged food and beverage products surveyed (*n* = 807/15,357) displayed HSR labels. The highest rates of uptake were for cereals, convenience foods, packaged fruit and vegetables, sauces and spreads, and ‘Other’ products (predominantly breakfast beverages). Products displaying HSR labels had higher energy density but had significantly lower mean saturated fat, total sugar and sodium, and higher fibre, contents than non-HSR products (all *p*-values < 0.001). Small but statistically significant changes were observed in mean energy density (−29 KJ/100 g, *p* = 0.002), sodium (−49 mg/100 g, *p* = 0.03) and fibre (+0.5 g/100 g, *p* = 0.001) contents of HSR-labelled products compared with their composition prior to adoption of HSR. Reformulation of HSR-labelled products was greater than that of non-HSR-labelled products over the same period, e.g., energy reduction in HSR products was greater than in non-HSR products (−1.5% versus −0.4%), and sodium content of HSR products decreased by 4.6% while that of non-HSR products increased by 3.1%. We conclude that roll-out of the voluntary HSR labelling system is driving healthier reformulation of some products. Greater uptake across the full food supply should improve population diets.

## 1. Introduction

Dietary guidelines generally recommend consumption of food groups that are minimally processed, and low in added sugars, trans and saturated fats and sodium [1]. However processed foods contribute approximately three quarters of dietary energy and nutrients consumed in many high-income countries [2]. Nutrition labelling is a recommended policy approach to promote and support healthier population diets [3,4,5]. Display of some form of nutrition information on pre-packaged foods is mandatory in many countries [6], and is often found on the back of food packaging. In recent years, a growing number of governments and organisations have begun adopting interpretive front-of-pack (FOP) labels that depict and interpret the nutrition content of a food using graphics, symbols or colours [7].

Interpretive FOP nutrition labels may guide healthier food choices [8,9], especially amongst more nutrition-conscious shoppers [10]. Importantly however, there is evidence suggesting that interpretive FOP labels also improve population diets through healthier product reformulation. Adoption of the Choices nutrition logo in the Netherlands [11], the Health Check Program symbol in Canada [12], and the Pick the Tick logo in New Zealand [13] and Australia [14] all led to improvements in the nutrient profile of food products on the market. However, these prior studies of reformulation effects largely comprised self-reported data on limited food groups from select food manufacturers, and focused mainly on sodium. To date, there has been no systematic and comprehensive assessment of the effects of interpretive FOP labels on food reformulation.

**Figure 1:**
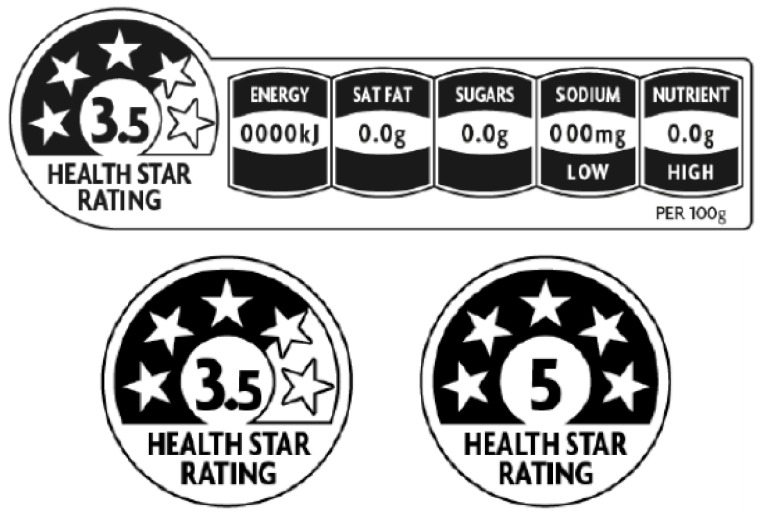
Example of Health Star Rating label (HSR) label formats.

In 2014, New Zealand and Australia adopted a new voluntary, interpretive FOP nutrition labelling scheme, the Health Star Rating (HSR). HSR rates the nutrition content of packaged food in half-star increments from half a star (least healthy) to five stars (most healthy) (Figure 1). The number of stars displayed is calculated based on the energy, saturated fat, total sugar, sodium, and fruit, vegetable, nut and legume (FVNL) levels and, in some instances, the protein, fibre and calcium content [15]. Our aim was to evaluate the effects of implementation of the HSR programme in New Zealand on product reformulation using data from annual systematic surveys of the labelling and composition of packaged foods and household panel food purchase records.

## 2. Materials and Methods

### 2.1. Study Design

We conducted an observational study of the composition of packaged foods before and after the introduction of the HSR system in New Zealand. Three independent annual surveys of packaged food labelling and composition were undertaken in supermarkets in 2014 (pre-implementation), and 2015 and 2016 (post-implementation). These data were linked with nationally representative household food purchasing data to obtain estimates of effects weighted by annual household food purchasing volumes.

### 2.2. Packaged Food Labelling and Composition Data

Since 2011, our team has collated data on mandatory back-of-pack Nutrition Information Panel (NIP) data for all packaged food and non-alcoholic beverages available in major New Zealand supermarkets (the Nutritrack database) [16]. Annual surveys are undertaken by trained fieldworkers to collect brand, barcode, nutrient, ingredient and front-of-pack labelling information for all packaged food and non-alcoholic products in four main supermarket stores in Auckland between February and April each year. The four store brands from which Nutritrack data are collected from represent the largest retail brands of the two main national supermarket retailers: Foodstuffs (54% grocery market share) and Progressive Enterprises (38% market share) [17].

Data are collected directly from all packaged products displaying a NIP Field workers use a custom-designed smartphone application (app) to scan product barcodes and photograph all surfaces of food packages. The aim is to collect information on the widest range of packaged products possible across store brands, with a view to generating a representative picture of the New Zealand packaged food supply. As such barcodes and product information are collected once only, i.e., if a barcode is collected in one store brand then information on that same barcode or product is not collected in a subsequent store. Different pack sizes of the same product are recorded as unique items in the database. Information is not collected for products that do not display a NIP including unpackaged fresh fruit and vegetables, fresh meat, and alcohol. Data are however collected for packaged fresh fruit and vegetables, and bulk buy items with a NIP seasonal products, such as Easter eggs, are excluded, as are dietary supplements including probiotics, vitamins, and minerals.

Photographs are used as source data to enter the following information into a secure, online database: Barcode, product name and brand, package size, recommended serve size, all mandatory values reported on the NIP, i.e., energy, protein, total fat, saturated fat, total carbohydrate, total sugars, and total sodium per 100 g or 100 mL (and other non-mandatory nutrient values, e.g., fibre, where reported on the NIP), ingredients, and front-of-pack labeling including HSR star graphic icons. Fibre values are only available for a minority of products because it is not mandatory to list fibre on the NIP Missing values are not imputed. Products are classified in a hierarchical structure into 15 food ‘groups’, 66 ‘categories’, and 183 ‘subcategories’ using a standardized system developed by the Global Food Monitoring Group [18], and used by the International Network for Food and Obesity/non-communicable disease Research, Monitoring and Action Support (INFORMAS) [19]. Quality assurance procedures include: One in every 10 products entered into the database is randomly selected for a full quality assurance check against product photos; regular reports are run to identify extreme or missing values for major nutrients; and the categorisation of all products is checked to ensure consistency within and between years. Any identified errors are corrected. If the accuracy rate of key fields drops below 98.8% a further 10% of products is selected for quality checking until the accuracy rate is >98.8%.

The Nutritrack database holds labelling and composition information for 14,368 items available in New Zealand stores in 2014, 14,415 items in 2015, and 15,358 items in 2016. Based on matching with products in the Food Switch smartphone app [20], which contains packaged food product information from all food retail stores in New Zealand, we estimate Nutritrack encompasses approximately 75% of packaged food and non-alcoholic products with NIPs nationally.

### 2.3. Household Panel Food Purchase Data

Nielsen Homescan^®^ is a geographically and demographically representative panel of approximately 2500 New Zealand households who scan all foods and beverages purchased for consumption in the home [21]. The Nielsen Homescan^®^ panel has been in existence for 20 years, and the weighted panel data represent approximately 80% of total national food and grocery retail business [22]. Homescan^®^ households are sampled randomly from metropolitan and non-metropolitan areas across three geographic regions in New Zealand (Upper North Island, Lower North Island, and South Island), and stratified by life stage (young families, older families, mixed families, young adults/singles, older adults/couples), household size, and household income. The target sample size is 2500 households but yearly sample size varies depending on number of households providing useable data. The sample is weighted to the New Zealand population using government statistics and census data, projected up to 1.6 million households [23].

Participating households are given barcode scanners, and household members are instructed to scan the barcodes on all purchased items on returning home after every shopping trip. Scanning occurs continuously throughout the year and includes products purchased from supermarkets, convenience stores, specialist stores, and department stores. Day, time, store, price, and promotion information is collected via the scanner. A barcode book is used to scan non-barcoded items such as fresh fruit and vegetables, fresh meats, delicatessen, and bulk bin items.

### 2.4. Data Linkage

Homescan^®^ data were obtained for 2014 (*n* = 1726 households and 4.65 million products), 2015 (*n* = 1827 households, 4.89 million products) and 2016 (*n* = 1839 households, 4.89 million products). Each product in the Nutritrack database was linked with the corresponding product in the Homescan^®^ database in order to weight results by annual household purchase volumes. Products were linked using barcodes and, where necessary, approximate string matching (which links products based on brand name, product name, and package size). If a product in Nutritrack could not be matched with a corresponding product from Homescan^®^, no data were available for household purchases of that product. Instances where products could not be matched were most likely due to changes in barcode following alterations to product packaging. All linkage was undertaken using standardised code across years in SAS statistical analysis software (Version 9.4, SAS Institute Inc., Cary, NC, USA).

### 2.5. Outcomes of Interest

Outcomes assessed for each year were numbers of products displaying the voluntary HSR star graphic label (uptake) by food group and food category; star ratings of products displaying a HSR label; nutritional composition per 100 g (energy, saturated fat, total sugar, sodium, protein, and (where available) fibre) of products displaying HSR labels compared with composition of those that did not display HSR labels; the composition of products displaying HSR labels in 2016 compared with the composition of the same products prior to introduction of the HSR label in 2014 (within-product reformulation); and changes in the composition of HSR-labelled products over time compared with changes in the composition of non-HSR-labelled products over the same period (2014–2016).

### 2.6. Data Analysis

HSR uptake, star ratings of products displaying HSR labels, and the nutritional composition of foods were summarized with the use of descriptive statistics for each of the three years separately. Weighted estimates were calculated by multiplying the nutrient content of each product by the number of units purchased, summing the resulting values for all foods and then dividing the sum by the total units purchased. Continuous variables were summarized as means and standard deviations (SD). The nutritional composition of products displaying HSR labels was compared with the composition of products that did not display HSR labels using independent sample *t*-tests. The nutritional composition of products displaying HSR labels in 2016 was compared with the composition of the same products in 2014 using paired *t*-tests. Changes in nutrient composition of HSR-labeled products over time were compared with changes in the composition of non-HSR products. Statistical analyses were performed in SAS version 9.4 software (SAS Institute Inc., Cary, NC, USA). All statistical tests were 2-tailed, and *p*-values < 0.05 were considered statistically significant.

## 3. Results

Of the total 44,141 products in the Nutritrack database (all three years of data), 35,602 (80.7%) were matched successfully to a product in the Nielsen Homescan database, thus providing household purchase volume data. Match rates were similar across all three years of data.

### 3.1. Uptake of the Voluntary HSR System over Time 

**Figure 2:**
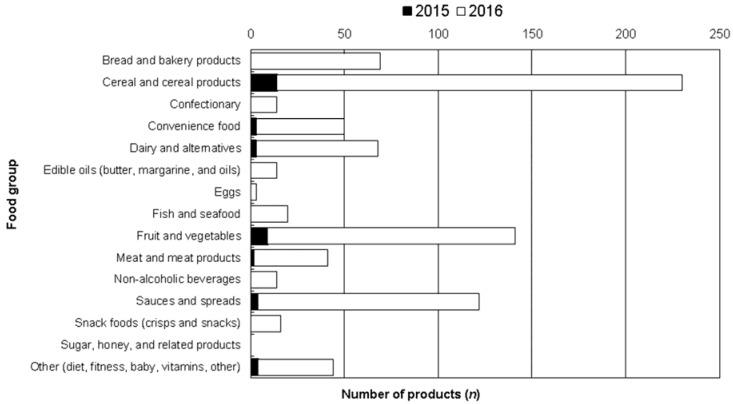
Number of products (*n*) displaying Health Star Rating (HSR), in 2015 and 2016. No products in the sugar, honey, and related products food group displayed a HSR label.

Nutritrack data are collected between February and April each year, and HSR was adopted as a voluntary initiative in June 2014. Therefore, no products for which information was collected in 2014 displayed the HSR label. In 2015, 39 of 14,415 (0.3%) products in the Nutritrack database carried HSR star graphics. In 2016, this increased to 807 of 15,358 products (5.3%). Figure 2 summarises uptake of HSR in 2015 and 2016 by food group. By 2016, five of the 15 food groups contained at least 50 products that displayed a HSR graphic. HSR availability weighted by annual purchases was 0.8% in 2015 and 7.2% in 2016, suggesting that HSR labels were on more frequently purchased products (data not shown).

### 3.2. HSR Star Ratings on Products in 2015 and 2016

**Figure 3:**
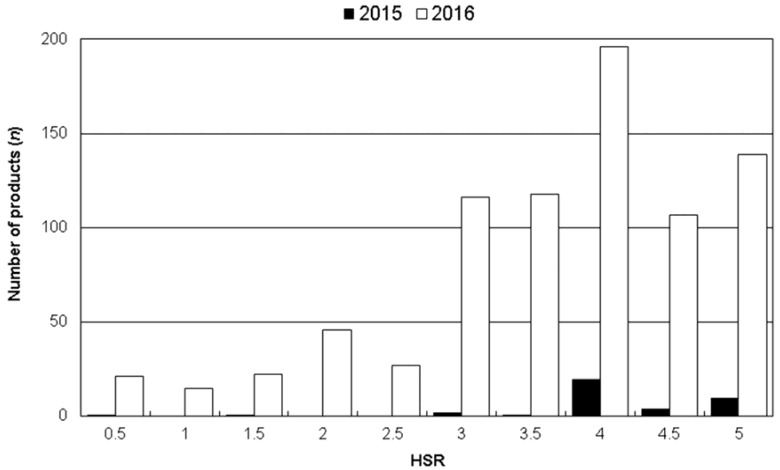
Number of products (*n*) displaying each Health Star Rating (HSR) (0.5 to 5 stars), in 2015 and 2016.

In 2015, 95% of HSR-labelled products (*n* = 37/39) had star ratings in the top half of the star range i.e., 3.0 to 5.0 stars (Figure 3). In 2016, the number of HSR-labelled products increased and the full range of ratings (0.5–5) were represented. Nevertheless, 84% of HSR-labelled products in 2016 displayed star ratings of 3.0 to 5.0 (*n* = 676/807), and only 131 (16%) displayed ratings in the 0.5 to 2.5 star range. 

For products that displayed the HSR star graphic in 2016 (*n* = 807), the median star rating was 4.0. Median star rating was 3.0 or higher in all food groups, other than confectionery (median 1.0). Dairy, Beverages, and ‘Other’ food groups all had median star ratings of 4.5 (data not shown).

### 3.3. Nutritional Composition of Foods by HSR Status

Table 1 summarises the nutritional composition of foods by HSR status in 2015 and 2016. The mean + SD energy content of products displaying HSR star graphics changed little between 2015 (1141 ± 857 KJ/100 g) and 2016 (1189 ± 812 KJ/100 g). The saturated fat, total sugar, and sodium contents of HSR-labelled products were higher in 2016 compared to 2015, which may reflect the increase in number and diversity of products displaying HSR star graphics in 2016, e.g., new food groups adopting the system that year included bread and bakery, snack foods, and confectionary. The fibre content of HSR-labelled products was also higher in 2016, whilst protein content was unchanged.

In 2016, products displaying HSR star graphics had higher energy density than products that did not display HSR labels (mean difference 119.2 KJ/100 g, 95% CI 63.7 to 174.8, *p* < 0.001). However, products displaying HSR labels were significantly lower in saturated fat (−2.1 g/100 g, 95% CI −2.6 to −1.8), total sugar (−2.3 g/100 g, 95% CI −3.4 to −1.4), and sodium (−217.5 mg/100 g, 95% CI −260.3 to −174.7) (all *p*-values < 0.001), compared with products not displaying HSR labels (Table 1). Such differences between HSR and non-HSR products could reflect reformulation, selective application of HSR to already healthier products, or a combination.

### 3.4. Reformulation of Products Displaying HSR Labels

In order to determine if the healthier profile of HSR versus non-HSR products was due to reformulation, a comparative analysis was undertaken of the nutritional composition of 431 products that displayed HSR star graphics in 2016 and were also available in 2014 (i.e., prior to adoption of HSR). For the purpose of this analysis, reformulation was defined as a minimum 5% change in at least one key nutrient (energy, saturated fat, total sugar, sodium, protein, or fibre). Under this definition, 356 (83%) of products displaying HSR star graphics in 2016 had been reformulated since 2014. The two major food groups where reformulation was evident were Cereals and Cereal Products (26% reformulated products) and Sauces and Spreads (20% reformulated products). Remaining food groups each accounted for <10% reformulated products.

Small but statistically significant changes were observed in overall mean energy (−29 KJ/100 g, *p* = 0.002), sodium (−49 mg/100 g, *p* = 0.03) and fibre (+0.5 g/100 g, *p* = 0.001) contents of the 431 products between 2014 and 2016 (Table 2). Changes in saturated fat, sugar and protein contents were not statistically significant. Estimates weighted by household purchase volumes displayed a similar pattern to the unweighted data, although effect sizes were slightly larger, suggesting that households were buying greater volumes of the reformulated HSR products than might be expected based on their proportional availability in-store.

**Figure 4:**
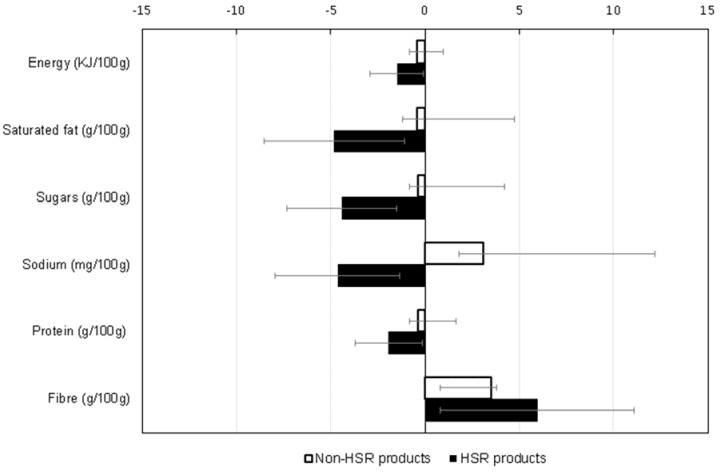
Percent changes in nutrient composition between 2014 and 2016 for Health Star Rating (HSR) and non-HSR products (estimates weighted by household purchase volumes). Note: Error bars indicate the 95% confidence intervals. Percent change calculated by estimating % change for each product between two years, and then estimating overall mean of all % changes. Fibre values were missing (not reported on the Nutrition Information Panel) for 209 HSR-labeled products (50%) and 5796 non-HSR-labeled products (77%).

A comparative weighted analysis of products that did not display HSR labels in 2016 and were also available in 2014 (*n* = 7505), showed that reformulation of HSR-labelled products was greater than that of non-HSR-labelled products, despite the healthier nutritional profile of the former at baseline (Figure 4). For example, the reduction observed in the energy content of HSR-labelled products was greater than that of non-HSR products (−1.5% versus −0.4%), and whilst sodium content of HSR-labelled products decreased by 4.6% the sodium content of non-HSR products actually increased (by 3.1%). Reformulation varied by food group and the observed decrease in energy density overall was largely driven by reductions in energy density of dairy foods and sauces, whilst the decrease in sodium overall was due mainly to reductions in the sodium content of cereals and convenience food (Table 3). 

## 4. Discussion

Two years following adoption of the voluntary front-of-pack Health Star Rating System in New Zealand, approximately 5% of packaged food and non-alcoholic beverage products displayed HSR star graphic labels. Food groups with the highest rates of uptake of HSR labels were cereals, convenience foods, packaged fruit and vegetables, sauces and spreads, and ‘other’ products (predominantly breakfast beverages). The majority of products displaying HSR labels had star ratings greater than 3.0, and the median rating was 4.0. Products displaying HSR star graphic labels had significantly lower mean saturated fat, total sugar and sodium contents, and higher fibre content, compared to non-HSR products. Approximately eight in 10 products (83%) displaying HSR graphics had been reformulated to some extent, and small but significant favourable changes were observed in mean energy, sodium and fibre contents, compared with product composition prior to adoption of HSR.

Our analysis used food labelling and composition data for more than 44,000 packaged food products available for sale in New Zealand over a three-year period spanning implementation of the Health Star Rating System. Rigorous, standardised methods were used for the annual survey data collections and a key strength therefore lies in comparing results over time (i.e., year on year). Linkage of these data with household food purchasing data enabling estimation of effects weighted by product purchase volumes was also a significant strength. 

Some limitations should however be considered in relation to the use of these data to monitor implementation of the HSR system in New Zealand. First, Nutritrack data collections take place in four supermarket stores in Auckland and may not capture products only available in other stores or other regions. Second, annual data collections take place February to April each year so analyses in this report do not include products that adopted HSR labels after April 2016. Third, fibre data were only available for a minority of products (*n* = 4230, 28% in 2016) because it is not mandatory to list fibre on the NIP in New Zealand. However, fibre is usually listed for products that have a sizeable content. Finally, because the number of products displaying HSR star graphic labels in the database was small (39 products in 2015 and 807 products in 2016), caution should be exercised in interpreting data and drawing conclusions pending wider uptake.

Manufacturers reformulate products continuously so it is possible that the changes we observed were due to global portfolio reformulation trends and not specifically linked to introduction of HSR. However our analysis comparing reformulation of HSR-labelled products with reformulation of unlabelled products indicates that HSR may have played a role. Furthermore, our findings are consistent with those of other studies of front-of-pack labelling systems and product formulation. Vyth et al. surveyed 47 Dutch food manufacturers who adopted the Choices nutrition logo (*n* = 878 products) and reported that uptake of the programme influenced manufacturers to reformulate existing products and develop healthier new ones, particularly products lower in sodium and higher in dietary fibre [11]. A similar Canadian study surveyed 14 Health Check program licensees representing 371 products and found that 150 products had their sodium content reduced in order to meet the criteria, leading to a total reduction of over 322,000 kg of sodium [12].

Prior to adoption of the HSR System in New Zealand and Australia, the best known front-of-pack interpretive labelling system in both countries was the National Heart Foundation Tick Programme. Analysis of the composition of breakfast cereals, breads and margarine products that were reformulated to meet the Tick criteria suggested that the programme led to removal of approximately 33 tonnes of salt from the New Zealand food supply in a one-year period [13]. A similar analysis in Australia of the effects of the Tick programme on the formulation of 12 breakfast cereals found that the average 40% reduction in sodium made to meet the programme criteria led to removal of 235 tonnes of salt annually from the national food supply [14]. The Heart Foundation of New Zealand recently announced that it was retiring the Tick programme, in part due to the roll-out of the government-endorsed HSR system [24].

### Implications for Policy

Although adoption of the HSR system is progressing steadily, star graphics are currently displayed on a very small proportion of packaged products in New Zealand. Continued adoption of the system by manufacturers and retailers will be essential to achieve wide penetration across the New Zealand packaged food supply and deliver meaningful benefits to population diets. Currently, the majority of HSR-labelled products have star ratings of 3.0 or higher, and relatively few display star ratings of 0.5 to 2.5. To optimise the impact of the system on consumer understanding and choices, it is important to achieve a balanced distribution of star ratings on products. If voluntary implementation fails to achieve widespread uptake by industry, consideration should be given to making the programme mandatory.

Early indications are that the HSR system incentivises healthier product reformulation, particularly sodium reduction and increased dietary fibre. Small but significant reductions in energy content were also observed. The positive effect of FOP labelling systems like HSR on healthier packaged food reformulation is encouraging, and particularly important in light of evidence that such systems have very limited effects on consumer behaviour and food choices [10,25]. However, far greater uptake of the HSR system by industry and adoption across the full breadth of the packaged food supply would be necessary for such reformulation to impact population diets in a healthful way. In addition, given marked concerns by consumers and public health practitioners about the sugar content of some products with high HSR ratings [26], reducing added sugar in HSR-labelled products should be an important focus for future industry reformulation efforts.

## 5. Conclusions

This comprehensive analysis of the effects of the Health Star Rating FOP nutrition labelling system on food reformulation in New Zealand shows that, two years post-implementation, the voluntary system is being adopted by industry and is effecting healthier product reformulation of some products. However, greater uptake of the system across the full breadth of the packaged food supply is essential if the observed product reformulation is to deliver meaningful benefits to population diets.

## Figures and Tables

**Table 1:** Nutritional composition of foods by Health Star Rating (HSR) status, in 2015 and 2016

Nutrient Content	2015				2016			
	HSR Products (*n* = 39)	Non-HSR Products (*n* = 14,376)	Mean Difference (95% CI)	*p*-Value *	HSR Products (*n* = 806)	Non-HSR Products (*n* = 14,526)	Mean Difference (95% CI)	*p*-Value *
	Mean (SD)				Mean (SD)			
Energy, KJ/100 g	1141.4 (856.5)	1065.9 (774.9)	75.5 (−174.7 to 325.5)	0.55	1189.3 (812.2)	1070.1 (777.2)	119.2 (63.7 to 174.8)	< 0.0001
Saturated fat, g/100 g	2.0 (3.1)	4.8 (7.5)	−2.8 (−3.8 to −1.7)	< 0.0001	2.9 (5.1)	5.0 (7.9)	−2.1 (−2.6 to −1.8)	< 0.0001
Total Sugars, g/100 g	7.3 (8.8)	14.0 (19.9)	−6.7 (−9.7 to −3.8)	< 0.0001	11.3 (13.5)	13.6 (19.6)	−2.3 (−3.4 to −1.4)	< 0.0001
Sodium, mg/100 g	204.2 (271.8)	506.6 (1771.5)	−302.4 (−398.8 to −206.1)	< 0.0001	276.5 (437.9)	494.0 (1812.2)	−217.5 (−260.3 to −174.7)	< 0.0001
Protein, g/100 g	8.9 (6.9)	7.2 (8.0)	1.7 (−0.9 to 4.3)	0.20	8.7 (8.3)	7.3 (8.1)	1.4 (0.9 to 2.0)	< 0.0001
Fibre, g/100 g	4.7 (3.8)	4.5 (9.3)	0.2 (−1.3 to 1.7)	0.78	5.8 (5.3)	5.6 (22.2)	0.2 (−0.7 to 1.0)	0.65

SD = standard deviation; 95% CI = 95% confidence interval. * Paired *t*-test on the mean (parametric). Fibre values were missing (not reported on the Nutrition Information Panel) for 12 HSR-labelled products (31%) and 10,753 non-HSR-labelled products (75%) in 2015, and 295 HSR-labelled products (37%) and 10,800 (74%) non-HSR labelled products in 2016.

**Table 2:** Composition of products displaying HSR labels in 2016 and composition of the same products in 2014 prior to introduction of HSR.

Nutrient Content	Unweighted Estimates (*n* = 431 Products)					Estimates Weighted by Household Purchase Volumes (*n* = 419 Products)				
	2014	2016	Mean Difference (95% CI)	*p*-Value *	% diff ^#^	2014	2016	Mean Difference (95% CI)	*p*-Value *	% diff ^#^
	Mean (SD)					Mean (SD)				
Energy, KJ/100 g	1028.8 (790.4)	1000.2 (782.0)	−28.6 (−46.5 to −10.6)	0.002	−1.5	1026.8 (794.6)	997.3 (785.8)	−29.5 (−48.0 to −11.0)	0.002	−1.5
Saturated fat, g/100 g	2.3 (3.7)	2.2 (3.7)	−0.1 (−0.2 to 0.01)	0.07	−3.7	2.3 (3.7)	2.2 (3.8)	−0.1 (−0.2 to 0.01)	0.07	−4.8
Total sugars, g/100 g	9.9 (11.7)	9.6 (11.3)	−0.3 (−0.7 to 0.1)	0.14	−4.6	9.8 (11.8)	9.5 (11.4)	−0.3 (−0.7 to 0.1)	0.18	−4.4
Sodium, mg/100 g	357.4 (627.7)	308.4 (441.7)	−49.0 (−93.0 to −5.0)	0.03	−6.7	363.2 (635.4)	312.7 (446.8)	−50.5 (−95.7 to −5.2)	0.03	−4.6
Protein, g/100 g	8.5 (8.7)	8.3 (8.6)	−0.2 (−0.9 to 0.4)	0.5	−1.9	8.2 (8.3)	8.0 (8.2)	−0.2 (−0.9 to 0.4)	0.48	−1.9
Fibre, g/100 g	4.9 (5.4)	5.4 (5.2)	0.5 (0.2 to 0.8)	0.001	5.3	4.6 (4.9)	5.1 (4.7)	0.5 (0.2 to 0.9)	0.001	6.0

SD = standard deviation; 95% CI = 95% confidence interval; Diff = difference.* Paired *t*-test on the mean (parametric); ^#^ Calculated by estimating % change for each product between two years and then estimating overall mean of all % changes.

**Table 3:** Composition of foods displaying Health Star Ratings (HSR) in 2016 compared with composition of the same foods in 2014 (prior to introduction of HSR), by food group.

HSR Status	Year	Total Number of Products in Category	Nutrient Content (Crude Mean (SD))						
			Energy (KJ/100)	Saturated Fat (g/100 g)	Sugars (g/100 g)	Sodium (mg/100 g)	Protein (g/100 g)	Fibre	
								*n*^ #^	g/100 g
**Bread and Bakery Products**									
Do not display HSR label	2014	32	1505.5 (344.6)	2.9 (3.7)	5.2 (8.9)	400.9 (151.4)	10.3 (14.8)	10	7.3 (6.9)
Display HSR label	2016		1534.4 (356.8)	2.6 (4.1)	5.1 (9.1)	406.6 (123.2)	7.9 (1.5)		4.7 (2.5)
Mean difference			28.9 (65) *	−0.3 (0.8)	−0.1 (2.4)	5.7 (105.1)	−2.4 (14.5)		−2.6 (6.8)
**Cereal and Cereal Products**									
Do not display HSR label	2014	105	1487.7 (358.4)	1.2 (1.7)	19.2 (12.1)	247.7 (171.8)	9.4 (4.3)	91	7.5 (5.4)
Display HSR label	2016		1481.2 (364.5)	1.2 (1.7)	18.6 (11.3)	228.3 (153)	9.3 (4.2)		8.1 (5.4)
Mean difference			−6.5 (89.9)	0.02 (0.3)	−0.6 (3.3)	−19.4 (41.1) *	−0.1 (0.8)		0.6 (1.8) *
**Confectionary**									
Do not display HSR label	2014	4	2020 (430.6)	9.7 (7.1)	57.2 (5.1)	71.8 (28.9)	5.4 (1.1)	1	0 (-)
Display HSR label	2016		2047.5 (389.5)	14.7 (10.1)	54.7 (5)	48.5 (41.4)	5.3 (1.7)		2.7 (-)
Mean difference			27.5 (41.9)	5 (6.4)	−2.5 (0.7) *	−23.3 (56.9)	−0.1 (1.1)		2.7 (-)
**Convenience Food**									
Do not display HSR label	2014	34	213.1 (95.9)	1 (1.6)	2.6 (1.4)	280 (65.2)	1.6 (0.9)	19	0.9 (0.8)
Display HSR label	2016		209.2 (98.1)	1.02 (1.4)	4.1 (10)	267.5 (53.5)	1.5 (1)		1 (0.6)
Mean difference			−3.9 (13.8)	0.02 (0.2)	1.5 (9.3)	−12.5 (27.2) *	−0.1 (0.4)		0.1 (0.3) *
**Dairy and Alternatives**									
Do not display HSR label	2014	43	384.8 (373.9)	1.9 (4.7)	6.1 (4.8)	126.4 (317.9)	4.5 (4.5)	28	1.1 (0.9)
Display HSR label	2016		372.1 (388.5)	1.8 (4.7)	5.9 (5.1)	127.1 (318.2)	4.52 (4.6)		1 (1)
Mean difference			−12.7 (32.4) *	−0.1 (0.2) *	−0.2 (1.1)	0.7 (28)	0.02 (0.08) *		−0.1 (0.6)
**Edible Oils (Butter, Margarine, and Oils)**									
Do not display HSR label	2014	7	2718.6 (746.6)	14.4 (2.4)	0.6 (0.5)	298.1 (306.8)	0.8 (0.3)	0	
Display HSR label	2016		2601.4 (799.2)	13.6 (2)	0.6 (0.5)	303.4 (315.6)	0.8 (0.3)		
Mean difference			−117.2 (169.9)	−0.8 (1.5)		5.3 (10.1)			
**EGGS**									
Do not display HSR label	2014	3	638 (0)	3.5 (0)	0.3 (0)	169 (0)	12 (0)	0	
Display HSR label	2016		638 (0)	3.5 (0)	0.3 (0)	169 (0)	12 (0)		
Mean difference									
**Fish and Seafood**									
Do not display HSR label	2014	15	549.9 (162)	0.9 (0.7)	0.8 (1.6)	445.3 (90.4)	19.1 (4.1)	0	
Display HSR label	2016		568.5 (163.4)	0.9 (0.7)	0.8 (1.6)	399.3 (107.1)	19 (4)		
Mean difference			18.6 (52.3)			−46 (133.3)	−0.1 (0.4)		
**Fruit and Vegetables**									
Do not display HSR label	2014	53	1047.7 (1075.9)	2.5 (3.7)	7.4 (6.7)	159 (183.9)	7.1 (6.7)	29	4.5 (2.4)
Display HSR label	2016		1043.7 (1060.7)	2.3 (3.6)	7.6 (7.1)	151.4 (176.5)	7.1 (6.7)		4.9 (2.1)
Mean difference			−4 (68.7)	−0.2 (0.5) *	0.2 (1.3)	−7.6 (60.3)			0.4 (1.7)
**Meat and Meat Products**									
Do not display HSR label	2014	24	736.9 (278)	2.7 (3.4)	2.7 (2.3)	621 (305.3)	17.1 (3.7)	5	1.6 (1.6)
Display HSR label	2016		741.3 (240.5)	2.6 (3.3)	2.2 (2.3)	644.6 (313.9)	16.9 (4)		5.5 (2.1)
Mean difference			4.4 (65.2)	-0.1 (0.3)	−0.5 (0.8) *	23.6 (105.7)	−0.2 (2.2)		3.9 (3.5)
**Non-Alcoholic Beverages**									
Do not display HSR label	2014	9	938.1 (815.9)	2.2 (2.1)	24 (20)	91.8 (73)	5.9 (5.4)	2	4.1 (5.8)
Display HSR label	2016		603.8 (644.4)	1 (0.5)	12.8 (8)	75.9 (78)	12.4 (24.3)		4.1 (5.8)
Mean difference			−334.3 (679.6)	−1.2 (2.2)	−11.2 (19.1)	−15.9 (36)	6.5 (21.8)		
**Sauces and Spreads**									
Do not display HSR label	2014	78	1002.2 (907.5)	3.5 (4.1)	8 (10.4)	771.7 (1298.8)	8.7 (11.3)	20	1.8 (3.2)
Display HSR label	2016		891.7 (876.4)	3.1 (3.9)	8 (10.8)	545.9 (859.8)	7 (9.3)		4.7 (2.8)
Mean difference			−110.5 (343) *	−0.4 (2)	−0.003 (2.7)	−225.8 (1068.1)	−1.7 (6.5) *		2.9 (3.1) *
**Snack Foods (Crisps and Snacks)**									
Do not display HSR label	2014	5	1890.6 (354)	1.8 (0.7)	7.2 (4)	932.2 (588.3)	5.1 (5.4)	0	
Display HSR label	2016		1902 (346.1)	1.5 (1.1)	6.4 (4.8)	832.4 (488.5)	5.1 (5.6)		
Mean difference			11.4 (57.7)	−0.3 (0.8)	−0.8 (1.7)	−99.8 (222)	0.02 (0.6)		
**Other (Including Diet and Fitness Products)**									
Do not display HSR label	2014	26	577.1 (451)	0.9 (1.3)	8.1 (0.9)	115 (94)	10.8 (12.4)	24	5.9 (8.2)
Display HSR label	2016		574.5 (452.5)	1 (1.3)	7.9 (1.7)	114.3 (94.2)	13.7 (18.2)		5.9 (8.2)
Mean difference			−2.6 (1.9) *	0.1 (0.3)	−0.2 (1.4)	0.7 (1.7)	2.9 (14.9)		0.04 (0.3)
**Total**									
Do not display HSR label	2014	438	1028.8 (790.4)	2.3 (3.7)	9.9 (11.7)	357.4 (627.7)	8.5 (8.7)	229	4.9 (5.4)
Display HSR label	2016		1000.2 (782)	2.2 (3.7)	9.6 (11.3)	308.4 (441.7)	8.3 (8.6)		5.4 (5.2)
Mean difference			−28.6 (189.9) *	−0.1 (1.2)	−0.3 (4.5)	−49 (464.1) *	-0.2 (6.7)		0.5 (2.4) *

Note: No products in the sugar, honey, and related products group displayed a HSR label. There was a total of 438 products carrying HSR graphics in 2016 that were also available in 2014. The number of barcode products available for analysis were reduced for different nutrients as some products, especially with multiple NIP’s, had nutrient information missing; SD: Standard deviation. * indicates mean difference is statistically significant at 5% level; ^#^ Number of products for which NIP information on fibre content was available.
